# Concrete Paving Slabs for Comfort of Movement of Mobility-Impaired Pedestrians—A Survey

**DOI:** 10.3390/ijerph19063183

**Published:** 2022-03-08

**Authors:** Magdalena Wojnowska-Heciak, Jakub Heciak, Adam Kłak

**Affiliations:** 1 Department of Landscape Architecture, Institute of Environmental Engineering, Warsaw University of Life Sciences—SGGW, ul. Nowoursynowska 166, 02-787 Warsaw, Poland; 2 Faculty of Civil Engineering and Architecture, Kielce University of Technology, al. Tysiąclecia Państwa Polskiego 7, 25-314 Kielce, Poland; adamklak@tu.kielce.pl

**Keywords:** concrete paver, mobility-impaired people, pavement characteristics, pedestrian comfort

## Abstract

People with locomotion difficulties encounter many barriers in a pedestrian environment. Pavement quality has been shown to substantially affect pedestrian satisfaction in general, and its optimal design may contribute to reducing the stigma put on people with impairments. Our research involved a survey assessing perception and attitudes towards pavement quality and characteristics for pedestrian comfort. The classic correlation of the variables based on contingency tables was used to analyse data and to test whether respondents’ perceptions depended on certain profile features. The completion of the statistical inference was the use of advanced algorithms of the correspondence analysis method. The relationships between the variables were assessed optionally using the chi^2^ test. The study results revealed desirable parameters and features of paving surfaces. People who use manual wheelchairs and declare limb loss prefer medium- and large-format concrete pavers with smooth finishing and no bevelling. People with skeletal abnormalities or peripheral neuropathy prefer concrete slabs and surfaces made of small- or medium-sized panels arranged in a regular pattern, made of smooth non-slip concrete with narrow joints. Further laboratory tests are necessary to investigate whether increased water permeability of pavements constructed on eco-friendly base layers can curb damage to the pavement surface and provide long-term durability.

## 1. Introduction

### 1.1. People with Motor Difficulties and Pedestrian Circulation in Public Space 

People with motor disabilities often experience mobility difficulties. The definition of disability proposed by the WHO (World Health Organisation) is a multidimensional term covering disability, activity limitations, and participation limitations, reflecting the interaction between the characteristics of a person’s body and the characteristics of the society in which they live [1]. Groups of people with motor disabilities (wheelchair/crutches users) feel discomfort when the quality of the pedestrian routes is inadequate [2].

However, mobility difficulties affect a much larger group of people. Health-related insufficient physical activity is a common problem among seniors [3,4], but it can also be a temporary situation for people in the prime of life [5]. Caregivers or assistants share the struggle of overcoming physical obstacles in the urban space (pushing wheelchairs, etc.). The stress level of parents/carers pushing pushchairs is comparable to that of disabled people [6,7,8]. Mobility difficulties among various social groups indicate the importance of a universal design of pavement surfaces to benefit all pedestrians in general. 

### 1.2. Issues Raised by People with Disabilities 

The problems usually result from inadequate sidewalk design or construction, poor maintenance, or even natural characteristics of the terrain. Obstructions such as uneven surfaces, litter, overgrown vegetation, kerbs without ramps [9], badly-sited lamp-posts, inadequate slopes [10,11], potholes, or bikes restrict the usability of a sidewalk and may lead to accidents [12]. Moreover, with aging, the main implications affecting walking cover the ability to maintain balance and negotiate obstacles [13]. The comfort level of sufferers from irregular body temperature or incontinence is further reduced by a lack of adjusted facilities, including toilets [12]. 

### 1.3. Urban Trees, Rainwater, and Pavements

Field observations indicate that some pavement damage occurs because of tree root interactions with the pavement [14,15]. Trees attempt to find good air and water conditions in the topsoil layer just beneath the surface [16,17]. The conflicts between trees and pavements often occur due to inadequate tree species (mature or fast-growing) [16,18,19], not enough soil volume for roots or space for the tree in general [16,18], hard-pan underneath topsoil or soil characteristics [20], shallow foundations underneath the sidewalk (limited or no base materials), shallow irrigation, and distances between the tree and pavement of less than 2.0–3.0 [16]. Pavement displacement is less likely to occur when permeable paving with a gravel layer is used. The gravel is a buffer between the pavement surface and roots in the subgrade, potentially minimising pavement damage and maintenance requirements [21,22,23].

On the other hand, the presence of trees in the city, including those growing along the pavements, has an impact on residents’ well-being [24,25,26,27,28,29,30,31]. Trees combat many civilisation diseases, alleviate social stress, and help build friendly and inclusive neighbourhoods [26,32,33]. People deprived of contact with nature are more prone to depression and reduced functional efficiency, which in turn can lead to a significant deterioration in their quality of life [34]. The availability of urban forest and street trees appears to be particularly important for people with disabilities and other people with mobility difficulties, offering an alternative environment for alleviating health disproportions [35,36,37].

There seems to be a conflict between keeping trees in the city and maintaining pavement quality. Permeable pavements with an adequate base layer may be a solution as a rapid increase in impermeable paved surfaces in urban areas may result in flash floods and economic costs [38,39]. However, known permeable paving, such as a mineral surface, may not aesthetically match the urban context. Therefore, universal ecological rainwater management solutions must be found for densely built-up areas [40,41,42]. With this complexity of the urban context, specific characteristics of pavements for water permeability and pedestrian comfort should be determined, together with the structure of the pavement base layer, ensuring a tree-root-friendly environment and water holding capacity.

### 1.4. Surfaces Friendly to People with Disabilities

In addition to dedicated solutions (tactile ground surface indicators—TGSI) for visually impaired people [43] or ramps for overcoming pavement edge differences, certain pavement features increase the comfort of people with mobility difficulty [44]. For example, specific paver arrangements are preferable in terms of safe vibration rates. Wheelchair users are exposed to vibration levels that may influence the spine, increasing the risk of deformities, LBP, and other musculoskeletal disorders [45,46]. One of such arrangements is a 90° herringbone pattern with a bevel of less than 6 mm. Preferred over the 45° pattern, this arrangement ensures safe exposure to vibrations [47]. Laboratory studies confirmed a difference in vibration exposure between those two options for both the manual and electric-powered wheelchairs (at 1 m/s, significant differences were found in peak accelerations between the seat and footrest) (*p* < 0.0001) and between the pavement surfaces (*p* = 0.004) [44]. However, there is still a research gap concerning other pavement features preferred by people with a disability.

The conventional approach to proper pavement design includes testing the level of stresses, strains, deflections, and deformations resulting from the quality of the subsoil [41,48]; materials used [49,50]; and weather conditions [51,52]. Nowadays, citizen science and public participation in creating new design solutions, especially those dedicated to public space, are becoming significant factors in pavement design [53]. Therefore, the need to examine the perception of pedestrians sensitive to pavement quality is the focus of our study. Furthermore, the lack of data allowing the optimisation of pavement parameters for all city residents and the enhancement of city inclusiveness for disabled residents, combined with the aspect of improving city infrastructure resilience to heavy rains and local flooding and increasing the number of trees in heavily urbanised areas [54], has led us to this study. 

Barriers encountered by people with mobility difficulties are still a rarely undertaken issue. The main research gap is the lack of an interdisciplinary research approach combing social inclusiveness for people with mobility difficulties, technical issues concerning rain-water resilience, and environmental issues related to trees’ root system protection. This study is the first stage of the more comprehensive research. This study is aimed at determining the optimal parameters of pavement surface in terms of pedestrian comfort and unhindered movement of people with a motor disability. Results of our previous study based on in-depth interviews and “walk-and-interviews” revealed that asphalt and concrete paving surfaces are preferred over mineral surfaces used in urban public parks by people with disabilities [55]. Therefore, we focused on concrete paving surfaces as being more practical in terms of installation and management and being capable of being interchanged with stone surfaces. 

We wanted to test which features of paving surface are crucial, the size, joints, or the pattern, for the comfort of pedestrian circulation (see Supplementary S1: questions 18–29). The primary research question is what parameters of the pavement increase the comfort of pedestrian circulation according to people with motor disabilities?

## 2. Materials and Methods

This study included the analysis of pavement perception of a target group of people with a motor disability (using a manual wheelchair, electric-powered wheelchair, crutches, or walking frame). This criterion enabled us to gather respondents who are particularly sensitive to the pavement quality. 

**Figure 1:**
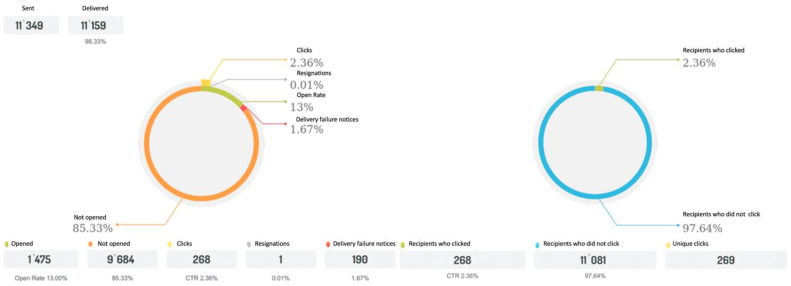
Survey recipients’ response to mailing (source: Avalon Foundation).

Our study involved a survey designed to assess perception and attitudes about pavement quality and parameters to ensure pedestrian comfort. To test the survey, we undertook a pilot survey among 15 respondents. The survey was voluntary and anonymous. The data were collected between 17 September 2021 and 17 October 2021. A link to the survey was distributed among 11,349 members of the Avalon Foundation [56] (Figure 1) via email and Facebook. The Avalon Foundation—Direct Help for the Disabled is a nationwide Polish foundation that works for the disabled and the sick. It is a non-profit, non-governmental public benefit organisation [56]. A total of 268 (2.36%) members opened the survey link, among whom 155 respondents participated in the study. The sample was restricted to individuals over 18 years old who use assistive devices (a manual or an electric-power wheelchair, crutches, or a walking frame) (Figure 1). 

According to the data of the Social Insurance Institution (in Polish: Zakład Ubezpieczeń Społecznych—ZUS, Poland), in December 2019, there were 2.4 million people in Poland receiving retirement and disability benefits, holding a certificate of disability or a certificate of incapacity for work [56,57,58,59], but there are no data on how many people are affected by a motor disability.

**Figure 2:**
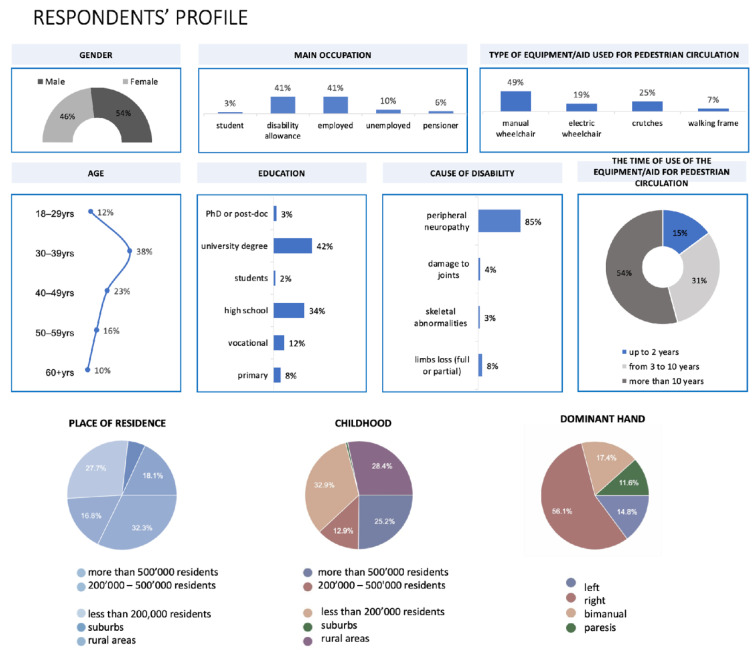
Respondents’ profile (*n* = 155) (source: processed by the authors).

The sample included people living in different types of places of residence: large (32.3%), medium-sized (16.8%), or small cities (27.7%); suburbs (5.2%); and rural areas (18.1%) (Figure 2). Most respondents suffered from damage to the nervous system (central or peripheral) or single or all muscle groups (85%). A total of 71 women (46%) and 84 men (54%) participated. Most respondents declared using a manual wheelchair (49%). More than half of the survey participants (54%) had been using assistive device users for more than 10 years.

**Figure 3:**
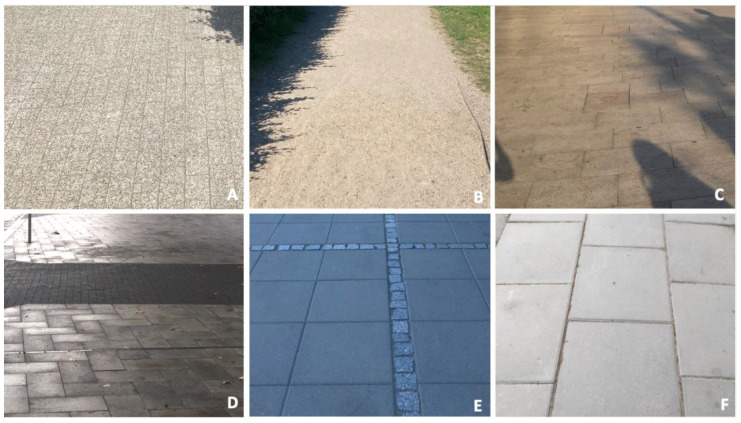
Various combinations of pavement surface subjected to perception analysis in the survey (**A**)—small-sized concrete paver, (**B**)—mineral surface, (**C**)—stone pavement, (**D**)—medium-format concrete paver, (**E**)—large-format concrete slab combined with granite paving pattern, F—large-format concrete slab. (source: processed by the authors).

The survey was based on a structured questionnaire with two main sections: (1) respondents’ profile and (2) questions concerning pavement features (Supplementary S1). First, we asked respondents about their gender, weight, height, age, education, causes of a motor disability, equipment/aids used for mobility, time of using the equipment, their dominant hand, the population of residence, and their primary occupation (Figure 2). In the second section, we asked about preferences towards full sun or shade while they walked on the pavement, as well as barriers encountered while walking. Participants responded using a 7-point Likert scale. The questions that followed referred to overgrown vegetation, unevenly placed paving blocks, and the frequency of facing these barriers. We then asked respondents about specific paving types in terms of pedestrian circulation comfort. We included photographs and descriptions of the pavement surfaces: A—small-sized concrete paver, B—mineral surface, C—stone pavement, D—medium-format concrete paver, E—large-format concrete slab combined with granite bricks, and F—large-format concrete slab (Figure 3). Another set of questions was focused on pavement details (bevels, patterns, shapes, and openings). Finally, respondents were asked about walking times on a particular type of pavement/path. 

The classic correlation of the variables according to the contingency tables was used to analyse data and test whether respondents’ perceptions depended on certain profile features. The completion of the statistical inference was the use of advanced algorithms of the correspondence analysis method. The relationships between the variables were assessed optionally using the chi^2^ test through the SPSS software.

Advanced algorithms in the manuscript were at the root of correspondence analysis. Correspondence analysis was based on factor analysis, the only difference being that it operated on qualitative variables. The degree of advancement of this analysis resulted from the inference method being similar to the factor analysis in which we rotated the created system to maximise the variance explained by the successive coordinates of this space. Moreover, it was based on the classical analysis of the multidimensional table in which the strength of the relationship between the variables was described using the chi^2^ statistic. Additionally, profiles were created on the basis of chi^2^ statistics, which enabled the determination of the number and strength of hidden relations in the input data set. Another complementary element that introduced advanced tools to the entire analysis was the creation of a script generating sequence charts based on Burt tables resulting from the correspondence analysis.

The assumed hypotheses were as follows:

**Hypothesis** **1** **(H1).** *The inadequate quality of the pavement is a common barrier for pedestrian circulation for people with a motor disability.*

**Hypothesis** **2** **(H2).** *Concrete paving is the most preferred solution for the comfort of pedestrian circulation, according to people with motor disabilities.*

The study was focused on a correlation between equipment/aid used for mobility and preferred pavement parameters by comparing data in cross-tables made in SPSS version 26.0 (SPSS, Inc., Chicago, IL, USA) and correspondence tables, diagrams, and graphs using Statistica software.

## 3. Results

### 3.1. Problems Resulting from the Inadequate Quality of the Pavement Is a Common Barrier in Pedestrian Circulation for People with Motor Disabilities

**Figure 4:**
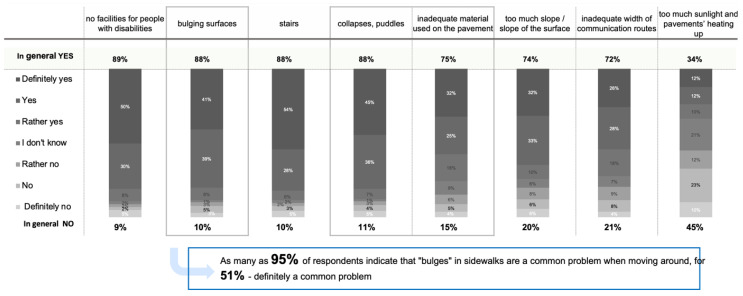
What problems do you most often encounter in public spaces? (source: processed by the authors).

The study confirmed hypothesis 1 (H1) that “inadequate quality of pavements is a common barrier in pedestrian circulation for people with a motor disability” (Supplementary S1: question 17 illustrated in Table 1; Supplementary S1: question 15 illustrated in Figure 4). Overall, 95% of respondents indicate that “bulges” in the pavement are encountered repeatedly (Supplementary S1: question 17 illustrated in Table 1). It should be noted that collapsing pavement (81%), bulges in the pavement (88%), and stairs (88%) were the most common inconveniences. Moreover, the lack of proper facilities was marked by 89% of the respondents. The type of material was indicated as an inconvenience by 75% of the respondents. The remaining set, inadequate width of pedestrian routes, inadequate slope, and the level of sunlight obtained <75% of indications by the respondents (Figure 4).

**Figure 5:**
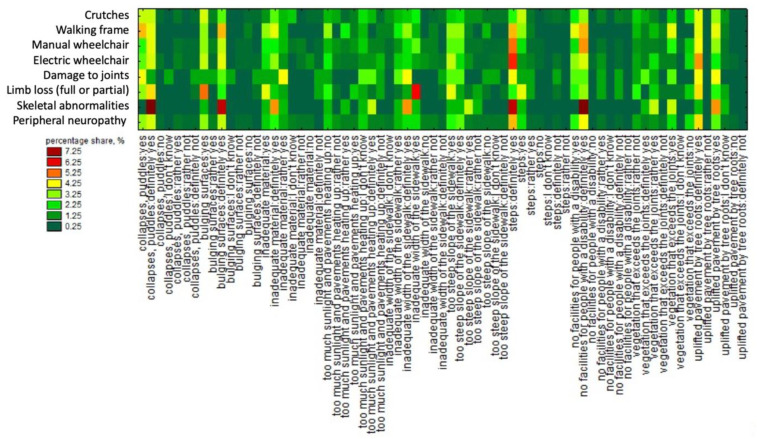
Sequence chart illustrating the numbers by type of a motor disability and the equipment used for pedestrian circulation and the most common pavement damage encountered (source: processed by the authors).

More information is provided by our sequence chart that shows the numbers according to the type of disability and the way of movement in a matrix. The results are shown in Figure 5. Figure 5 illustrates that stairs are the main barrier for pedestrians who use wheelchairs and walking frames. Bumps and collapses in the surface were often indicated as an inconvenience for people moving with the help of a walking frame. This type of pavement damage was also stated by people using an electric-powered and manual wheelchair who were to a large part affected by skeletal abnormality. The lack of facilities for people with a disability was also an inconvenience that was often indicated, especially by people who utilised a manual wheelchair or a walking frame and those who declared skeletal deformations. Grass and weeds in the joints or exposed tree roots and the lack of sun exposure were not regarded as important factors.

### 3.2. Pavements Made of Concrete Are the Preferred Type of Comfortable Pedestrian Path 

**Figure 6:**
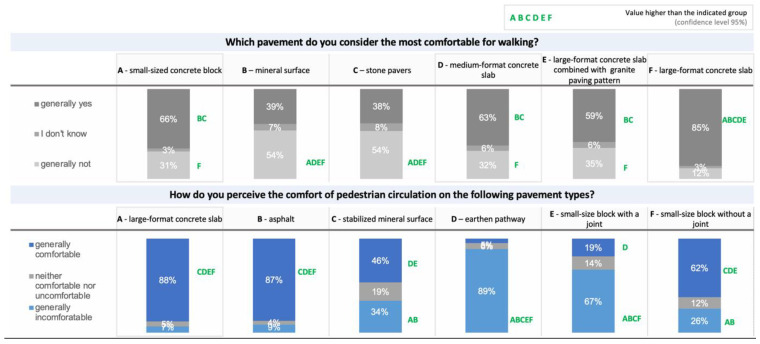
Which type of pavement do you consider to be the most comfortable for walking? (source: processed by the authors).

The study confirmed hypothesis 2 (H2) that “concrete pavement is preferred in terms of comfortable pedestrian circulation by people with a motor disability”. In addition, the results indicated that large-format concrete slabs were the most desirable (Figure 6).

**Figure 7:**
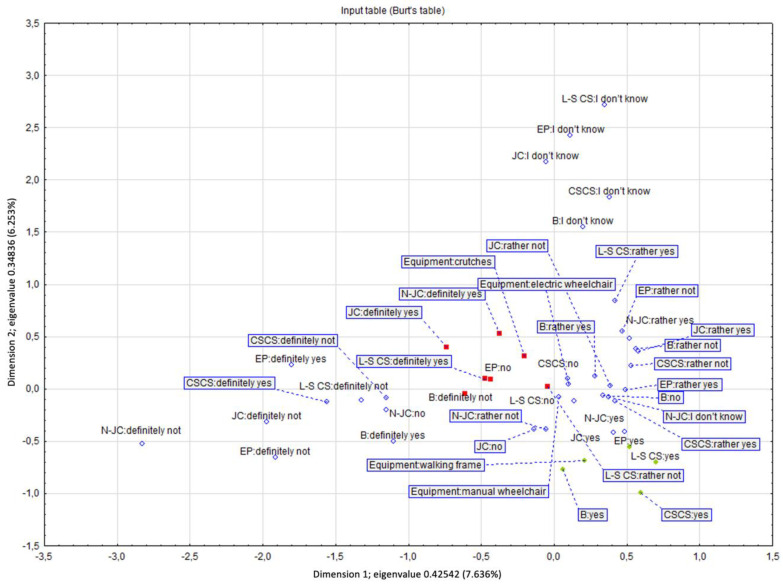
Correspondence analysis (source: own work). Key: large-format concrete slab (L-S CS), small format jointless concrete slab (N-JC), asphalt (B), small format slabs with joints (JC), stabilised mineral surface (CSCS), earthen pathway. Key: Colours indicate on grouping depending on the type of equipment used for moving with the preferences of the given surface: blue—wheelchair (manual or electric); red—crutches; green—walking frame.

The multidimensional analysis of the correspondence based on Burt’s tables allowed for the drawing of additional conclusions (Figure 7). The chi^2^ statistic <0.001 suggests a relationship between preferences regarding the type of pavement, type of disability, and the manner of moving around. Dimension 1 determines the hidden relationship between the variables and divides the strength of preferences for a given surface vs. the type of disability, while dimension 2 discriminates the type of pavement vs. the type of mobility aid, but additionally introduces additional discriminatory power in the form of eliminating respondents who showed indecision when indicating their preferences regarding the type of pavement. The first two dimensions with the highest inertia were used in the analysis. The first dimension divides a given feature according to acceptance level, separating the respondents’ feelings about the moving comfort on a given pavement type. It also demonstrates the respondents’ opinions by placing their strong preferences on the right. In the most extensive grouping of effects, less decisive responses dominate. The second dimension separates the response space regarding the type of equipment/aid used. For example, the green colour marks the respondents using walking frames who prefer the large-format concrete slabs (L-S CS), small jointless concrete pavers (N-JC), and asphalt (B). On the other hand, people using crutches prefer small format slabs with joints (JC), small jointless concrete pavers (N-JC), and large concrete (L-S CS) surfaces but do not like asphalt (B) or earthen pathways.

Interestingly, people who use manual and electric-powered wheelchairs have an undefined profile, which means they have a high level of acceptance for many types of surfaces. Therefore, particular focus should be placed on surfaces common to all groups (assistive equipment used). In this case, from the point of view of ease of movement, optimal surfaces are jointless large-format slabs (L-S CS) and small format jointless concrete pavers (N-JC) (Figure 7).

**Figure 8:**
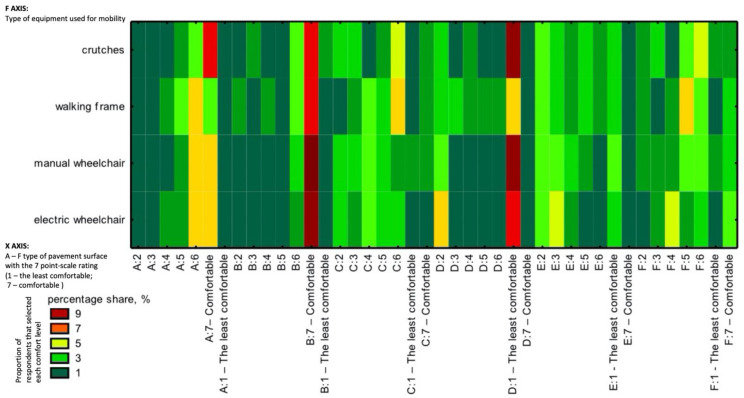
Sequence chart illustrating the comfort of pedestrian circulation (source: processed by the authors). Key: (**A**) large-format concrete slab, (**B**) asphalt, (**C**) stabilised mineral surface, (**D**) earthen pathway, (**E**) small format slabs with joints, (**F**) small format jointless concrete slab.

Important findings were obtained from a series of answers concerning the comfort of pedestrian circulation on a given type of pavement. The results are presented in a sequence chart (Figure 8). Analysis of the sequence chart (Figure 8) results show that people who use crutches prefer large-format concrete slabs. The orange colour in the chart, standing for the above-average number of respondents moving also in wheelchairs or with walking frames, indicates a high level of comfort perceived on large concrete slabs. Large concrete paving slabs are thus considered very appropriate pavement surfaces. All respondent groups strongly reject the earthen pathways, judging them as the least comfortable for movement. An interesting observation is the high rating of asphalt, which dominates as comfortable surface (Figure 8).

The respondents declared that large concrete paving slabs and small jointless concrete slabs were convenient. This result was obtained, regardless of the disability or equipment used to move around. It should be added that this conclusion was evident despite the large proportion of people representing one type of disability (damage to the nervous system). From the observations above, it follows that large concrete paving slabs are the most preferred pavement surfacing type. 

### 3.3. Characteristics of Optimal Concrete Pavement

**Figure 9:**
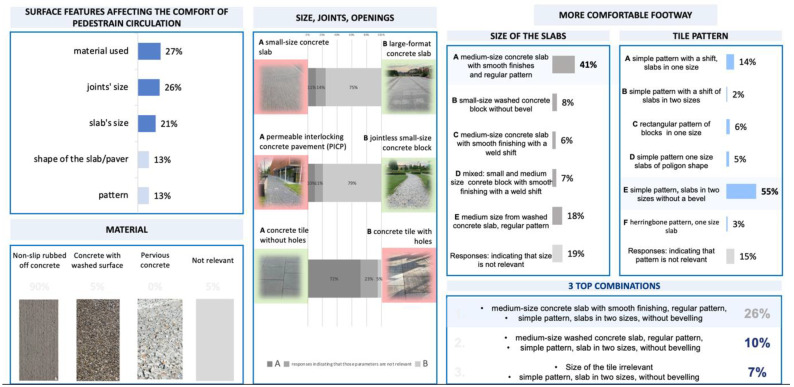
Evaluation of the optimal pedestrian surface features (source: processed by the authors).

The respondents indicated several pavement characteristics and parameters that significantly impact the comfort of moving (material used, slab size, pattern, joints, openings) (Figure 9). The results show the need to design an optimal pavement using smooth anti-slip concrete surfacing and to carefully consider the paving slab size and widths of joints or other openings so that they do not adversely affect pedestrians (i.e., vibrations).

**Figure 10:**
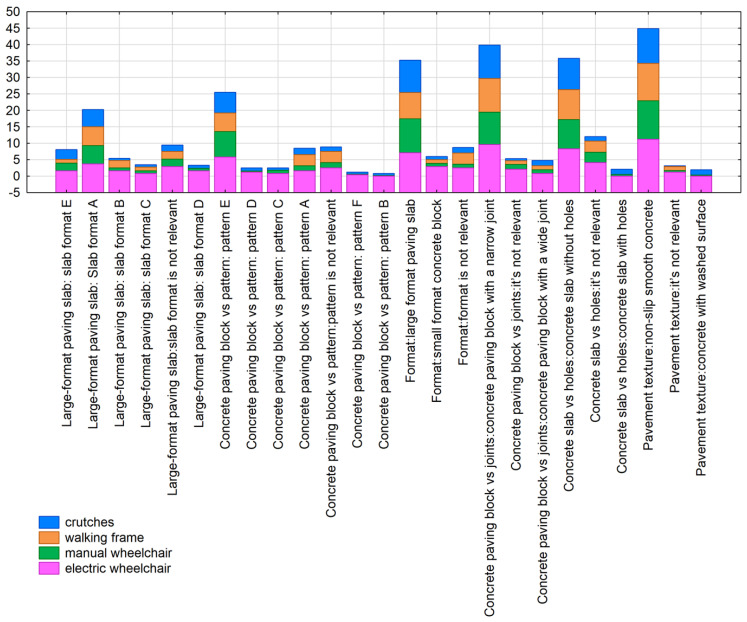
Percentage analysis grouped according to the comfort of pedestrian circulation on a concrete pavement (source: processed by the authors).

The respondents’ opinions on specific pavement parameters are crucial for hypothesis 2 “concrete pavement is preferred in terms of comfortable pedestrian circulation by people with a motor disability”. First, an analysis of the contingency table was performed, considering the effect of the equipment/aid used for pedestrian circulation (Figure 10). The percentages grouped according to moving comfort on the concrete pavement showed several solutions regardless of the equipment used, which facilitates the formulation of conclusions. It should be emphasised that a large percentage of the respondents who took a position regarding the comfort of pedestrian circulation on a given pavement are people using walking frames and manual wheelchairs. The following aspects of concrete pavement dominate:

1. Pavement texture: smooth non-slip concrete.
2. Concrete slabs with a narrow joint.
3. Concrete slabs without openings.
4. Large paving slabs.
5. A slab with simple patterns, two sizes, no bevelling.
6. A medium-sized concrete slab with smooth finishing and a regular pattern.

**Figure 11:**
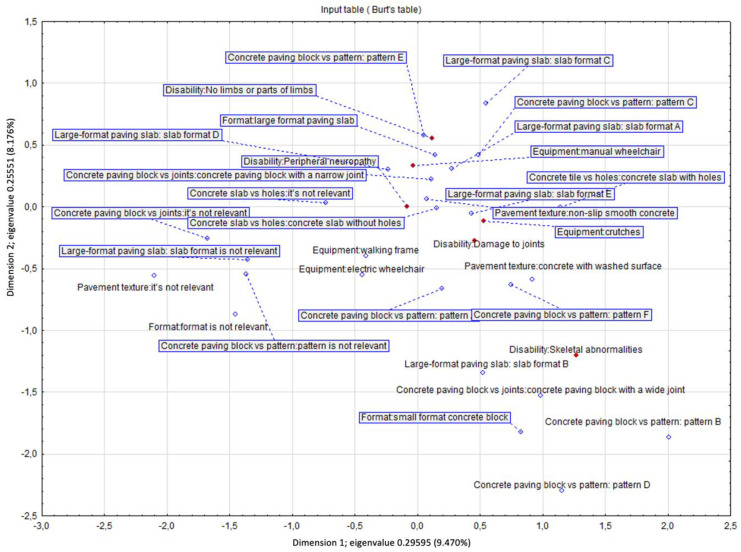
Correspondence analysis (source: processed by the authors).

Only the two most important dimensions (with the highest inertia of the variables) were used in the further analysis. The chi^2^ test <0.001 suggests a significant relationship between the equipment/aid used and the preferred features of the concrete pavement. The grouping results show that the first dimension discriminated/distinguished concrete pavement characteristics, indicating the lack or presence of impact of a given feature on the comfort of movement. The second dimension turned out to be more important and divided the respondents by the assistive devices they used. As a result, we found that people using manual wheelchairs and declaring limb loss prefer medium-size and large concrete slabs with smooth finishing, without bevelling. On the other hand, people using walking frames and electric-powered wheelchairs prefer concrete slabs and other surfaces made of small- or medium-sized slabs with a regular pattern made of smooth non-slip concrete with narrow joints (Figure 10). The correspondence analysis of contingency tables is presented in the chart below (Figure 11).

**Figure 12:**
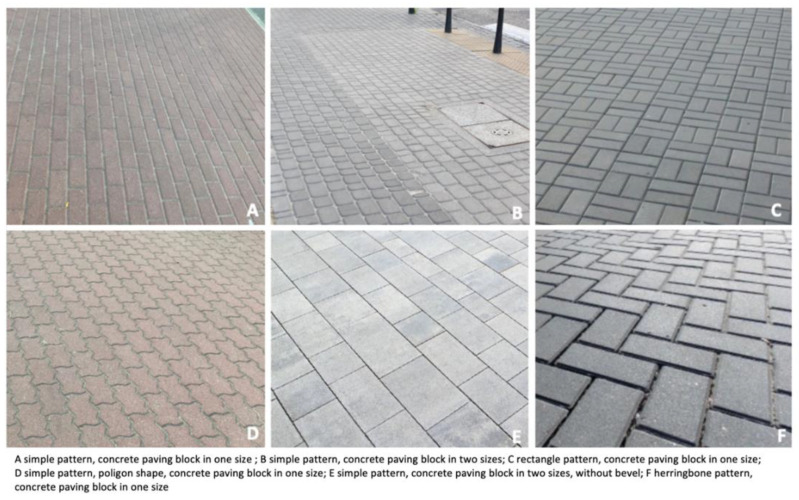
Concrete paving block patterns (**A**–**F**) analysed in correspondence with the analysis in Figure 11 (source: processed by the authors).

Figure 11 complements Figure 10 but is projected onto a multidimensional space resulting from a dozen variables. Figure 11 has X-values on the negative scale due to the reference frame adopted by the updated program. This does not change the results. There is the same distance between the points. Figure 12 illustrates the concrete paving patterns analysed in correspondence with the analysis in Figure 11. 

## 4. Discussion

Social inclusion in universal design refers to “a process that enables and empowers a diverse population by improving human performance, health and wellness, and social participation” [60]. It makes life easier, healthier, and friendlier for all [61]. The ongoing discussion over the actions that cities need to take to address the challenges related to city inclusiveness, defined, i.e., in the Sustainable Development Goals (SDGs): 10 Reduced Inequalities, 3 Good Health and Well-Being, 13 Climate Action, and 15 Life on Land [62], covers the issues of accessible pavements and other types of public spaces. However, most publications regarding pavement technical matters concern roads. Therefore, technical problems of pavement surfaces are rarely undertaken.

The study results revealed various barriers city residents with motor disabilities encountered in terms of movement comfort and pavement quality (Figure 3 and Figure 4). Given the damaging impact of stigmatising attitudes towards disability [63,64,65,66,67], a good quality pavement that facilitates the comfort of pedestrian circulation puts people with motor disabilities on an equal playing field [68,69,70,71]. The presence of people with disabilities in public space is not related to the total number of the people with impairments in that city, but with how the cities are adjusted to them, potentially serving as evidence that no obstacles are preventing them from being within the society [10].

Some barriers, including uneven pavement surface (Figure 3 and Figure 4), are related to other issues, such as rainwater infiltration in urban areas. Others refer to pavement bulges (Figure 3 and Figure 4) and are associated with urban trees growing along the pavements and roots penetrating the soil below pavements searching for water and air [16,17]. Our study results (Figure 5, Figure 6 and Figure 7) are consistent with the walking surface aspects indicated in the literature as significant to the comfort of pedestrian circulation [44,47] such as a slab format and pattern, the material used, or the presence of openings. In addition, the study results allow for the determination of the specific pavement characteristics to create universal design solutions according to the preferences of people with motor disabilities (Figure 8).

However, our research indicates contradictory expectations towards pavements. Asphalt surface, indicated by as a comfortable for movement by the respondents (Figure 8), due to the need to use a heavy equipment for the application, cannot always be used. Street trees planted along the footways prevent the use of that solution. Respondents declared the need to keep vibration rates low and to avoid collapses and bulges in the pavement. They suggested using smooth large-format concrete slabs without openings, preferably narrow joints, and no bevelling (Figure 8). This type of pavement is difficult to reconcile with expectations to keep pavements flat in the long term. Collapses and bulges are often related to precipitation or the presence of street trees. Permeable pavements and wide joints can reduce the damage [21,22].

We carried out our study during the fourth wave of the COVID-19 pandemic, and for health and safety purposes, the survey was conducted on a sample of internet users. Therefore, the model may not represent the target population, but it certainly shows the attitudes of specific groups of people with motor disabilities. 

As the municipalities or the Statistical Office provide no data concerning the number of manual/electric wheelchair users or people using crutches or “walking frames”, we cooperated with the largest foundation (the Avalon Foundation) for people with all types of disabilities in Poland. The mailing report from the Avalon Foundation states that our survey was sent to over 11,000 people on their mailing list with a response rate of 2.36% (Figure 1). Therefore, we may assume that we gathered a representative sample of respondents from our target group. 

Correspondence analysis was implemented to search for hidden relationships in qualitative traits. Of course, correspondence analysis only suggests some relationships between the variables within the examined set, which are difficult to detect with other methods. It is a tool that allows for the finding of specific relationships between the analysed variables. In the classical multidimensional analysis, information is obtained only regarding the strength of the relationship between the qualitative variables. Correspondence analysis is based on these assumptions, but introducing a metric (based on the chi^2^ test) provides us with information about the structure of relationships between the columns and rows of the multi-way table of the data set. It can also prioritise the strength of hidden effects in the relationship between the rows and columns of multi-way tables. It is similar to factor analysis but provides information on categorical variables. Hill developed detailed assumptions in 1974 [72].

## 5. Conclusions

Asphalt and concrete pavements and earthen pathways used for pedestrian circulation are the most and the least comfortable, respectively. However, asphalt surface does not always match the context of all public spaces (e.g., city squares, historic tree alleys). Moreover, the necessity of using heavy equipment to install asphalt makes its use sometimes impossible, especially in places with already existing trees. Therefore, we resigned from testing this solution in detail. Concrete being the “stone of the 20th century” and the most popular material used for pedestrian surfaces (for its price and availability) was chosen as the dominant subject of the study. However, it may be in many cases used interchangeably with stone surfaces. During the study, we obtained data that allowed for the description of the desirable parameters and features of comfortable paving surfaces indicated by people with certain types of mobility difficulties.

There is a continued need to address the barriers faced by people with motor disabilities. The response to discriminatory pavement deficiencies needs to be improved in terms of the accessibility of various public spaces and services offered by cities to mobility-impaired people.

Further research should therefore focus on field/laboratory tests of pavement characteristics defined in our study, improved in terms of water permeability features combined with the appropriate pavement base structure. Furthermore, additional tests are needed to analyse the vibration level and water permeability. 

This study is significant in providing the evidence that there are certain pavement surface features that impact on comfort of walking. The findings may be potentially applicable to other cities, as urbanites worldwide continue to find ways to convince residents to pedestrian circulation and resign from cars. 

## Figures and Tables

**Table 1:** Are “bulges” in pavements a common obstacle in pedestrian circulation? (source: processed by the authors).

	Definitely No	No	Rather Not	I Don’t Know	Rather Yes	Yes	Definitely Yes	Yes	Overall Yes	Overall No
“Walking frame”					9.1%	45.5%	45.5%	100.0%	100.0%	0.0%
Crutches		2.6%			13.2%	36.8%	47.4%	100.0%	97.4%	2.6%
Electric wheelchair		3.3%			6.7%	30.0%	60.0%	100.0%	96.7%	3.3%
Maunal wheelchair	1.3%	2.6%	2.6%		6.6%	36.8%	50.0%	100.0%	93.4%	6.6%
Overall	1%	3%	1%		8%	36%	51%	100.0%	95%	5%

## Supplementary Materials

The following supporting information can be downloaded at: https://www.mdpi.com/article/10.3390/ijerph19063183/s1, Supplementary S1: Questionnaire; Supplementary S2: Raw Data Analysis.
