# Supplementary material for: Concrete Paving Slabs for Comfort of Movement of Mobility-Impaired Pedestrians—A Survey
Source: Int J Environ Res Public Health. 2022 Mar 8;19(6):3183. doi: 10.3390/ijerph19063183 (PMC8950068; doi:10.3390/ijerph19063183)
Supplement: Supplementary file 1 [file ijerph-19-03183-s001.zip › ijerph-1579624-supplementary.pdf]

Questionnaire - Parameters of comfortable pedestrian surfaces

Section 1 Introduction

Good morning ,
We would like to invite you to participate in a study on issues related to the comfort of walking he pedestrian routes . The survey is aimed at adults in a wheelchair , " walking frame " or "he crutches ", as they are particularly sensitive to the quality of these spaces . The aim of the test is this determine the optimal parameters of the pavement , which ensures comfort of movement .
The survey results are collected anonymously and will only be statistically analyzed . Participation in the survey is voluntary , you can Stop answering at any time . It will also approximately 15 minutes this complete the survey . The form mainly consists of single or multiple choice closed questions . Mandatory questions are marked with a red asterisk .

Thank you for your time .

Section 2 Respondent's profile

1. Gender * (single choice)
   - Female
   - Male

2. Weight * (single choice)
   - 0-34kg
   - 35-54kg
   - 55-64kg
   - 65-74kg
   - 75-85kg
   - > 85kg

3. Height * (single choice)
   - <150
   - 151-160cm
   - 161-170cm
   - 171-180cm
   - > 180

4. Age * (single choice)
   - 18-25yrs
   - 26-35yrs
   - 36-50yrs
   - 51-65yrs
   - 66-75yrs
   - 76 + yrs

5. Education * (single choice)
   - Primary
   - vocational
   - High school
   - University degree

- Phd or post- doc
- student

6. Your physical disability results from : * (single choice)
    - limb loss
    - peripheral neuropathy individual or entire muscle groups (cerebral palsy, Heine-Medina disease, polyneuropathy)
    - skeletal abnormalities (achondroplasia, dwarfism, rickets)
    - damage to joints (joint Charcot , rheumatic diseases)

7. The most frequently used equipment for mobility * (single choice)
    - manual wheelchair
    - electric wheelchair
    - "walking frame"
    - crutches

8. The time you use the assistance in moving (wheelchair, walking frame, crutches) * (single choice)
    - less than 1 year
    - 1-2 yrs
    - 3-5yrs
    - 6-10yrs
    - > 10yrs

9. Dominant hand: * (single choice)
    - left
    - right
    - bimanual
    - paresis

10. Main life activity: * (single choice)
    - working
    - not working
    - student
    - pensioner
    - disability allowance

11. Place of residence * (single choice)
    - city with over 500,000 residents
    - city from 200,000 up to 500 thousand residents
    - city below 200,000 residents
    - suburbs
    - rural area

12. The place where you spent / you childhood * (single choice)
    - city with over 500,000 residents
    - city from 200,000 up to 500 thousand residents
    - city below 200,000 residents
    - suburbs
    - rural area

Section 3 Parameters of the surface of pedestrian routes * (single choice)

The questions in this section will be related to the comfort of walking on various types of pedestrian surfaces.

13. Do you prefer a walk in the shade or in a sunny place in the summer? * (single choice)
- shady place
- sunny place

14. Do you prefer a walk in the shade or in a sunny place in winter? * (single choice)
- shady place
- sunny place

15. What problems do you most often encounter in public spaces? * (single choice)

7-point scale Likert
- definitely not
- not
- probably not
- I do not know
- I think so
- Yes
- Definitely yes

- collapses, puddles
- bulging surfaces
- unsuitable material used on the pavement
- too much sunlight and pavements heating up
- inadequate width of communication routes
- too much slope / slope of the surface
- stairs
- no facilities for people with disabilities

16. Does the presence of vegetation overgrowing by joints affect the comfort of walking on the pavement? (7 point scale Likert) (single choice)

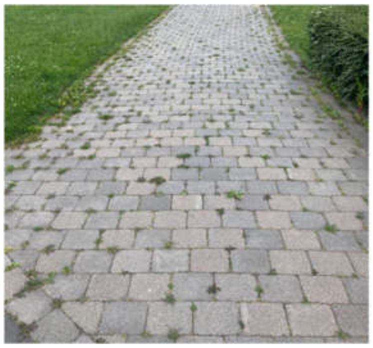

Pavement joints overgrown with vegetation (photo by M. Wojnowska-Heciak)

17. Are "bulges" in pavements a common obstacle in moving for you? (7 point scale Likert) (single choice)

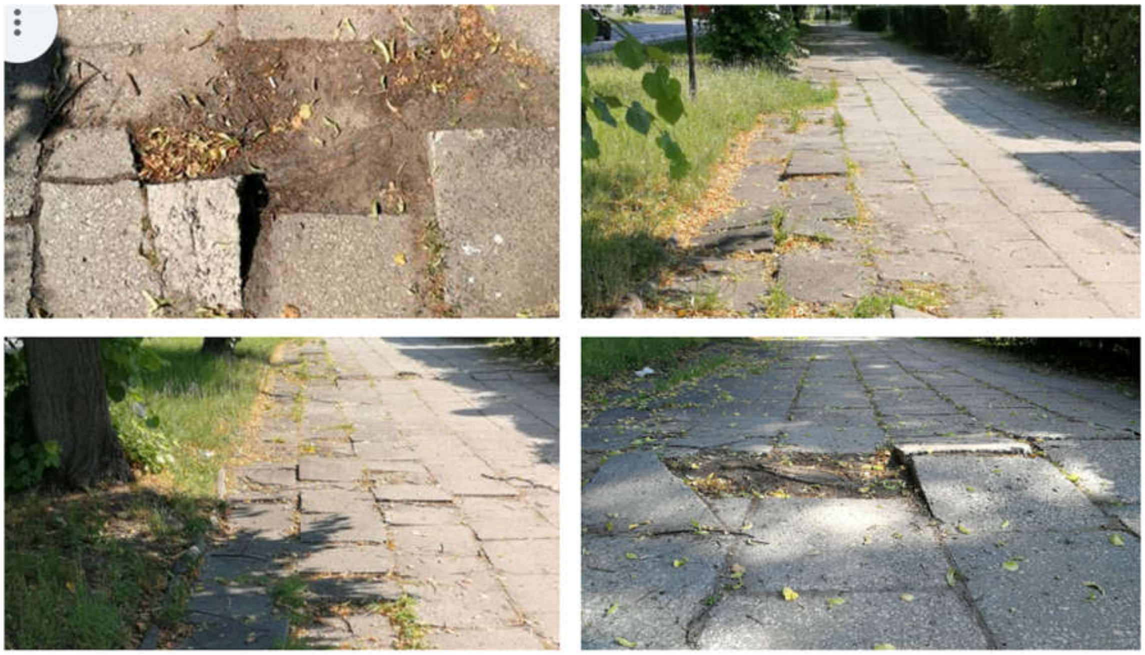

Sidewalks distorted by tree roots (photo A. Kłak)

18. Which of the surfaces do you consider to be the most comfortable for you to move about? (7 point scale Likert) (single choice)

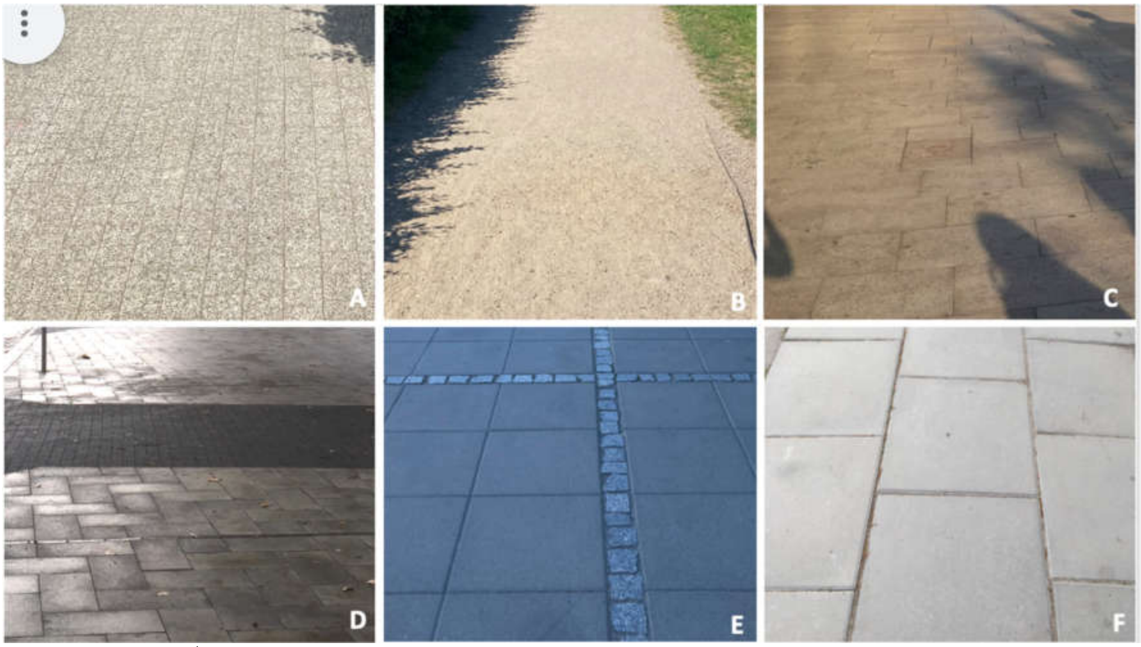

Type of material (A - small-sized concrete block, B - mineral surface, C - stone surface, D - medium format concrete slab, E - large-format concrete tile and granite cube, F - large-format concrete slab) elaboration M. Wojnowska-Heciak

- A
- B
- C
- D
- E.
- F.

19. Please justify your answer (open question)
………………………………………………………………

20. Which of the concrete surfaces do you consider the most comfortable for you to travel on? (single choice)

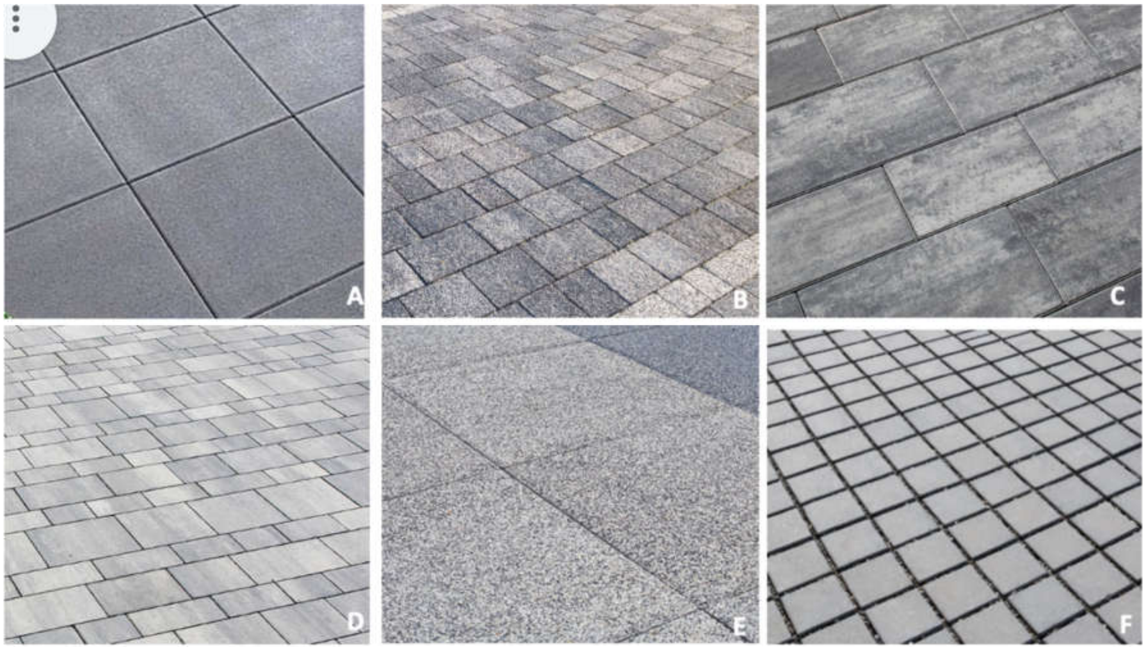

Size, pattern, roughness (A - medium format concrete slab with regular arrangement, B - small-size cube without washed concrete phase C - medium format concrete slab with a joint shift, D - mixed fine and medium-sized system with a joint shift without a bevel, E - medium format concrete slab with regular arrangement, made of washed concrete F - small-size concrete block with a wide joint) elaborated M. Wojnowska-Heciak

A- medium format concrete slab with regular arrangement,
B- small-sized cube without washed concrete phase
C- medium format concrete slab with a joint shift,
D- mixed fine and medium-sized system with a weld shift without a bevel,
E- medium format concrete slab with regular arrangement, made of washed concrete
F- small-sized concrete cube with a wide joint
does not matter

21. Please justify your answer (open question)
…………………………………………… ..

22. Please select the three most important parameters of the surface that affect the comfort of movement? (single choice)

- the material used (brick, concrete, stone, gravel, etc.)
- the size of the joints
- tile size
- tile / cube shape
- arrangement (straight, herringbone etc.)

23. Which of the concrete surfaces do you consider the most comfortable for moving around? (single choice)

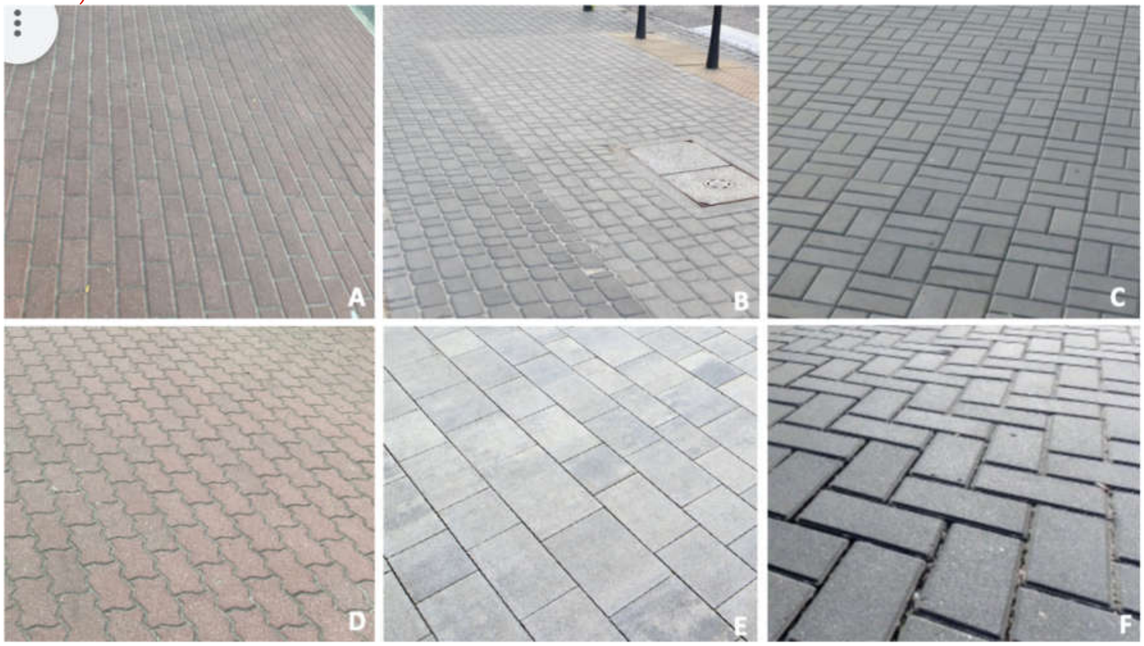

Various patterns and stacking methods cubes M. Wojnowska-Heciak

- A simple pattern with a shift, a cube in one size
- B simple pattern with an offset, cubes in two sizes
- C rectangular pattern, one size cube
- D simple pattern, one size cube, polygon shape
- E simple pattern, a cube in two sizes, without a bevel
- F herringbone pattern, one size cube
- Does not matter

24. Which of the concrete surfaces do you find more comfortable to move around? (single choice)

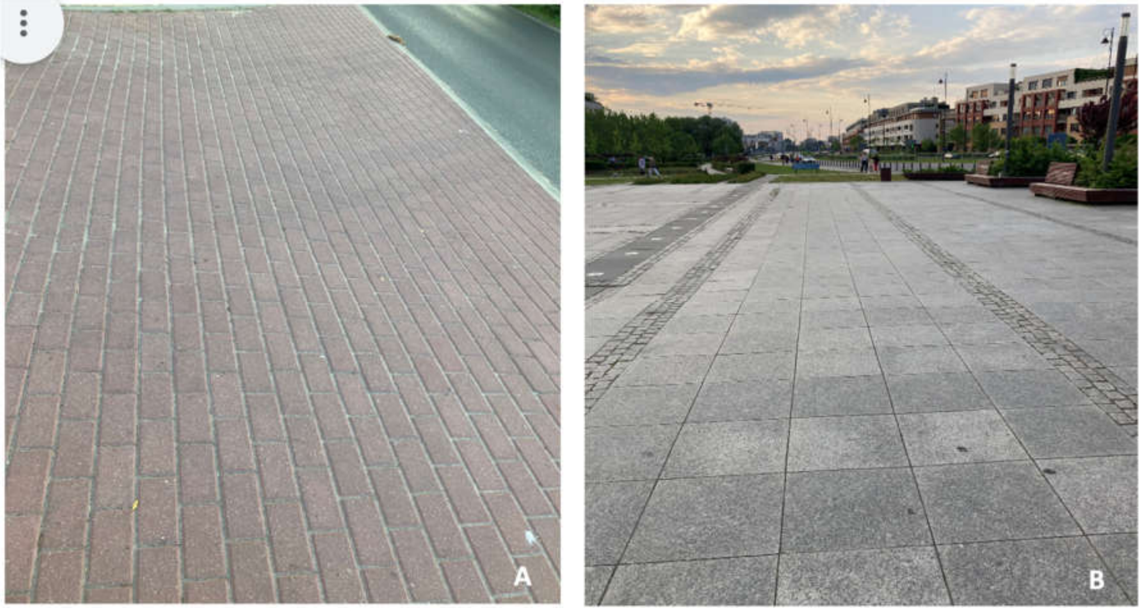

Different sizes of paving slabs (A - small-size concrete block, B - large-size paving slab) (photo by M. Wojnowska-Heciak)

- A - small-size concrete cube,
- B - large-format paving slab
- Does not matter

25. Which of the concrete surfaces do you find more comfortable to move around? (single choice)

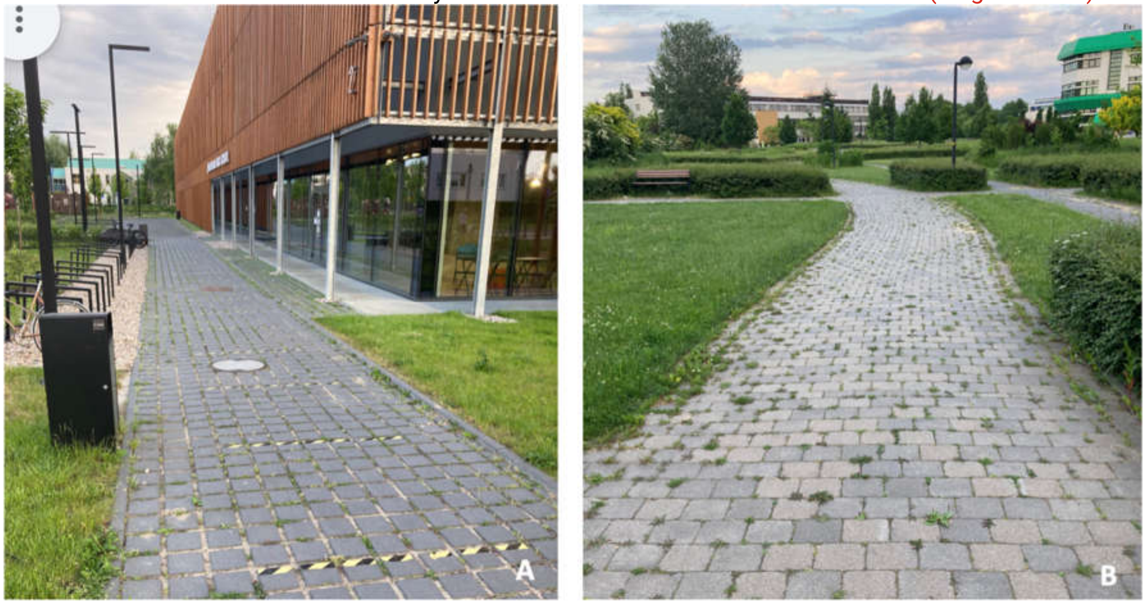

Various width of the joints (A - concrete cube with a wide joint, B- concrete cube with a narrow joint) (photo M. Wojnowska-Heciak)

- A concrete cube with a wide joint
- B concrete cube with a narrow joint
- Does not matter

26. Which of the concrete surfaces do you find more comfortable to move around? (single choice)

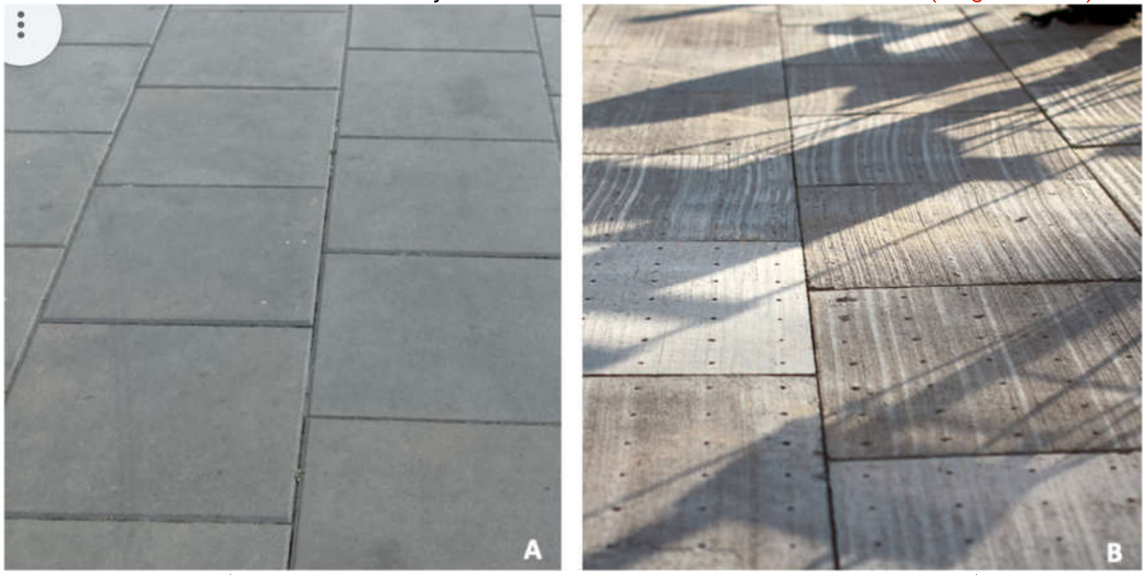

Openings in the tile (A - concrete slab without openings, B - concrete slab with openings) elaboration M. Wojnowska-Heciak

- A - concrete slab without holes,
- B - concrete slab with holes

- Does not matter

27. Which of the concrete surfaces do you find more comfortable to move around? (single choice)

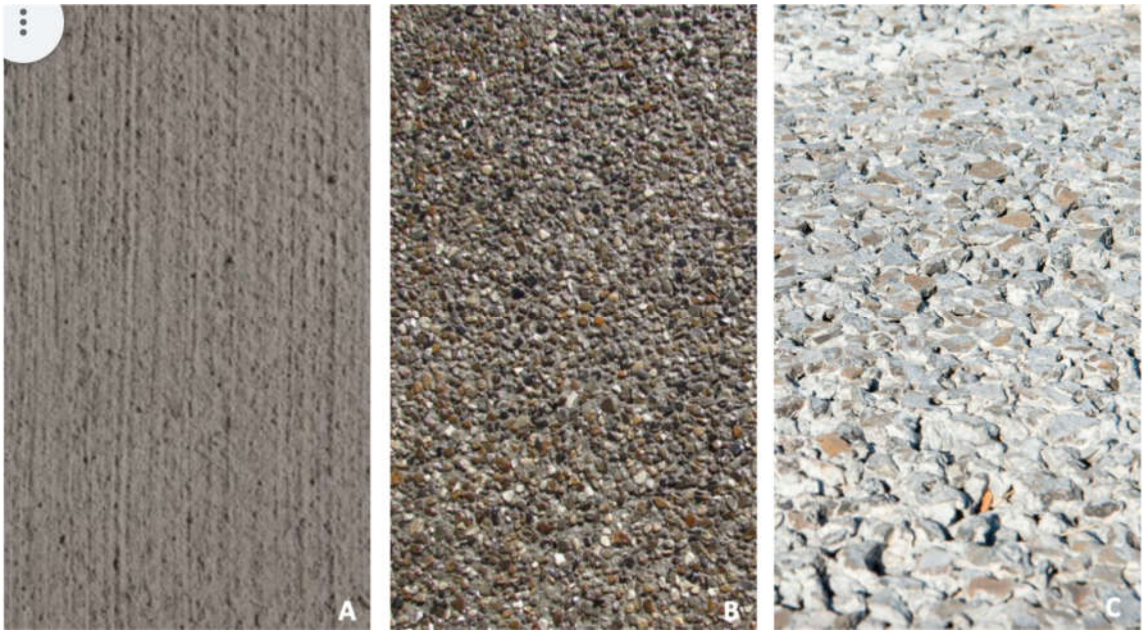

A smooth, non-slip concrete
- B concrete with exposed aggregate
- C pervious concrete (water-permeable)
- does not matter

28. How do you assess the comfort of moving along a given route in terms of comfort (1 - the least comfortable; 7 - the most comfortable)?

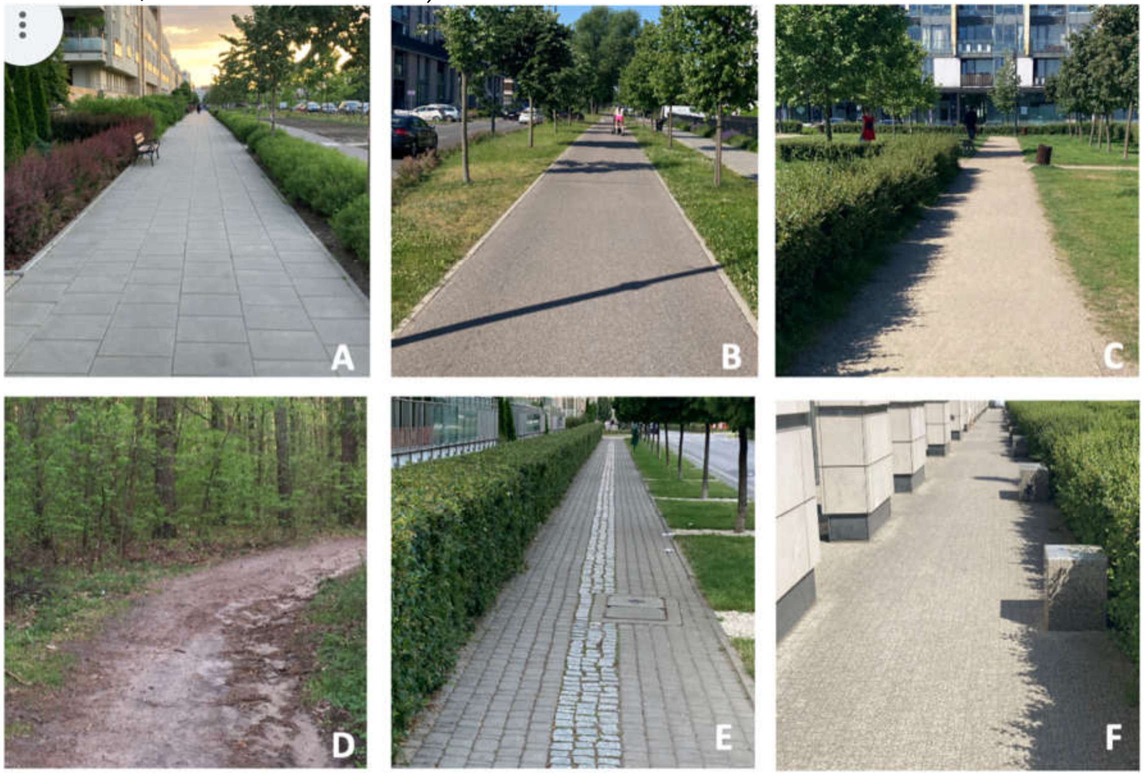

A - large-format concrete slab
B - asphalt
C - stabilized mineral surface
D – earthen surface
E - small-size cube with a joint
F - small-size cube without a joint

Scale: 1 - the least comfortable - - 7 - the most convenient

29. How long can you use a given surface without feeling discomfort related to moving?

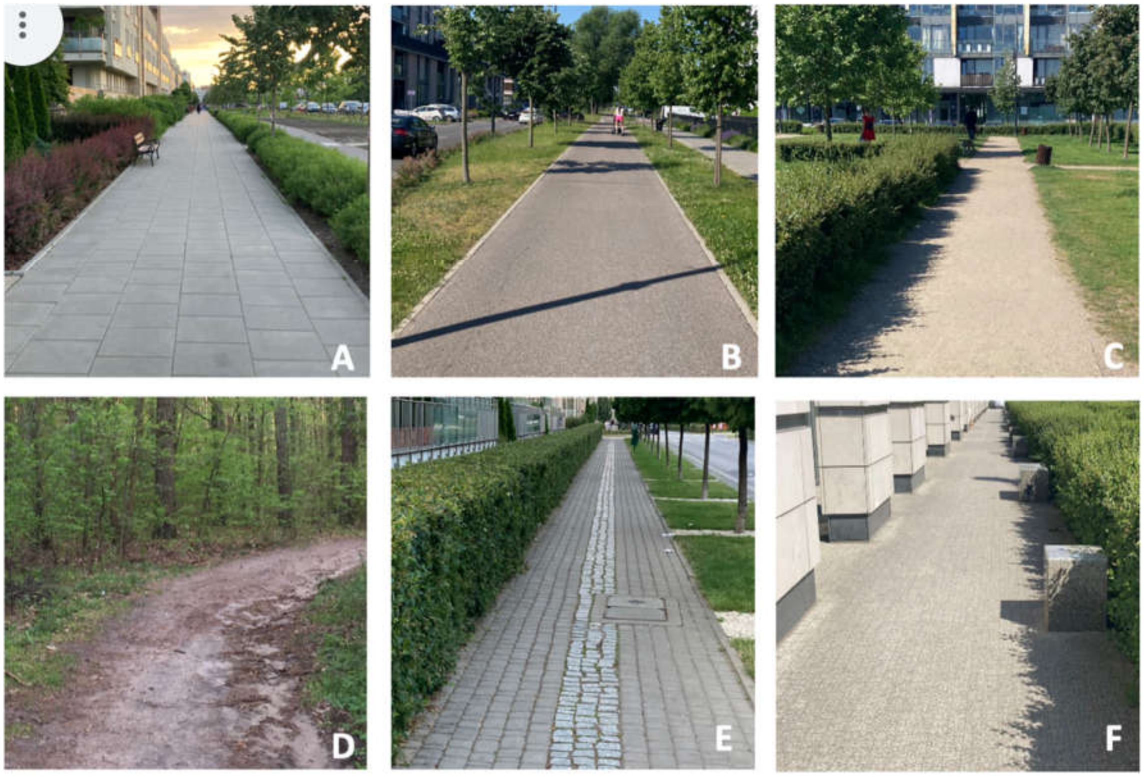

A - large-format concrete slab
B - asphalt
C - stabilized mineral surface
D - earthen surface
E - small-size cube with a joint
F - small-size cube without a joint

< 15 min
16-30 minutes
31-60 min
61-90 min
91-120 min
> 120 min
